# Unraveling the AMPK-SIRT1-FOXO Pathway: The In-Depth Analysis and Breakthrough Prospects of Oxidative Stress-Induced Diseases

**DOI:** 10.3390/antiox14010070

**Published:** 2025-01-09

**Authors:** Guangqi Guan, Yaoxing Chen, Yulan Dong

**Affiliations:** College of Veterinary Medicine, China Agricultural University, Haidian, Beijing 100193, China; gqguan@cau.edu.cn (G.G.); yxchen@cau.edu.cn (Y.C.)

**Keywords:** AMPK-SIRT1-FOXO pathway, oxidative stress, reactive oxygen species, antioxidant mechanism

## Abstract

Oxidative stress (OS) refers to the production of a substantial amount of reactive oxygen species (ROS), leading to cellular and organ damage. This imbalance between oxidant and antioxidant activity contributes to various diseases, including cancer, cardiovascular disease, diabetes, and neurodegenerative conditions. The body’s antioxidant system, mediated by various signaling pathways, includes the AMPK-SIRT1-FOXO pathway. In oxidative stress conditions, AMPK, an energy sensor, activates SIRT1, which in turn stimulates the FOXO transcription factor. This cascade enhances mitochondrial function, reduces mitochondrial damage, and mitigates OS-induced cellular injury. This review provides a comprehensive analysis of the biological roles, regulatory mechanisms, and functions of the AMPK-SIRT1-FOXO pathway in diseases influenced by OS, offering new insights and methods for understanding OS pathogenesis and its therapeutic approaches.

## 1. Introduction

Oxidative stress (OS) refers to a state of imbalance between oxidative and antioxidant mechanisms in the body. Under normal physiological conditions, the body’s oxidative and antioxidant systems maintain a dynamic equilibrium. When the body encounters external stimuli or internal metabolic dysregulation, this equilibrium is disrupted, resulting in the excessive production of reactive oxygen species (ROS) and free radicals, which cause cellular and tissue damage [1]. Over recent years, oxidative stress has become a major focus in health research. This review aims to provide a comprehensive analysis of the AMPK-SIRT1-FOXO pathway, focusing on its roles in oxidative stress, its regulatory mechanisms, and its therapeutic potential in combating diseases associated with oxidative stress, such as cancer, cardiovascular disorders, neurodegenerative diseases, and diabetes. Numerous studies indicate that OS is intricately linked to the development of a wide range of diseases, including cancer [2], cardiovascular disease [3], liver disease [4], neurodegenerative disorders [5], and diabetes [6]. Additionally, oxidative stress is strongly associated with the aging process, and it is considered a primary contributor to age-related decline [7].

AMPK is a central regulatory factor in cellular energy metabolism. Under conditions of oxidative stress, intracellular energy levels shift, leading to an increase in the AMP/ATP ratio, which activates AMPK. Activated AMPK then promotes various energy-regulating processes, such as enhancing fatty acid oxidation and increasing glucose uptake, to restore cellular energy homeostasis. In addition to its role in energy balance, AMPK also responds to oxidative stress by modulating downstream signaling pathways. For instance, AMPK phosphorylates PGC-1α, upregulating Nrf2 and thereby improving mitochondrial function, which enhances cellular resilience against oxidative damage and alleviates hyperlipidemia-induced fatty liver disease [8].

SIRT1 is a NAD+-dependent deacetylase that plays a significant role in the oxidative stress response. Changes in NAD+ levels induced by oxidative stress impact SIRT1 activity. By deacetylating various proteins, SIRT1 contributes to cellular antioxidant responses. For example, it deacetylates FOXO3, activating it to mitigate oxidative stress and prevent cell death [9].

FOXO, a key transcription factor, is modulated by several factors under oxidative stress. Through phosphorylation, AMPK enhances FOXO’s transcriptional activity, promoting antioxidant gene expression. FOXO regulates genes involved in antioxidant defense, such as superoxide dismutase (SOD) and catalase (CAT), thereby boosting cellular antioxidative capacity. Additionally, FOXO manages cellular processes like autophagy and apoptosis, helping to maintain cellular stability during oxidative stress [10]. Together, these factors play crucial roles in the antioxidant response. Studies have demonstrated that interventions like hydrogen-rich water can activate the AMPK-SIRT1-FOXO3a pathway, which mitigates oxidative stress and mitochondrial dysfunction, effectively easing conditions such as Alzheimer’s disease [11]. In summary, the AMPK-SIRT1-FOXO pathway is integral to oxidative stress regulation and represents a promising avenue for therapeutic approaches to various oxidative stress-related diseases.

Additionally, this review emphasizes recent advances in understanding how the AMPK-SIRT1-FOXO pathway contributes to energy homeostasis, mitochondrial quality control, and cellular adaptation, highlighting its potential as a target for novel therapeutic interventions. By consolidating the current knowledge, this review seeks to explore not only the fundamental mechanisms of the AMPK-SIRT1-FOXO pathway but also its broader implications for addressing oxidative stress-induced pathologies and aging-related conditions

## 2. Response Mechanism of Oxidative Stress

### 2.1. Generation Mechanism

#### 2.1.1. Enzymatic Reaction

NADPH oxidase plays a crucial role in ROS production across various cell types. Upon activation, NADPH oxidase transfers electrons from NADPH to oxygen molecules, generating superoxide radicals (O_2_^−^), which further react to form other reactive oxygen species (ROS) like hydrogen peroxide (H_2_O_2_) and hydroxyl radicals (OH) [12]. In addition to NADPH oxidase, other enzymes, including xanthine oxidase (XO), peroxidase (POD), and nitric oxide synthase (NOS), contribute to ROS generation under specific conditions [13]. For example, during ischemia–reperfusion injury, XO activity increases significantly, catalyzing the conversion of hypoxanthine to xanthine and uric acid, which leads to the production of large amounts of O_2_^−^ and H_2_O_2_ [14].

In inflammatory conditions like chronic colitis, myeloperoxidase (MPO) activity rises notably, producing superoxide radicals and other ROS that contribute to DNA damage and mutations, particularly in colon tumors [15]. Under physiological conditions, NOS generates nitric oxide (NO), which serves as a signaling molecule. However, in pathological contexts, NOS can produce excessive NO, which reacts with O_2_^−^ to form peroxynitrite (ONOO^−^), a potent oxidant that induces lipid peroxidation, disrupts mitochondrial function, and causes DNA strand breaks, ultimately triggering programmed cell death [16].

#### 2.1.2. Mitochondrial Dysfunction

Mitochondria are the primary sites for cellular energy production and a major source of ROS. Under normal physiological conditions, mitochondria generate energy through the electron transport chain (ETC), producing water as a byproduct along with small amounts of ROS [17]. However, during pathological states, such as aging or hypoxia, mitochondrial ETC function is impaired, leading to increased electron leakage and an overproduction of ROS. This heightened ROS production contributes significantly to cellular oxidative stress and subsequent damage [18]. Additionally, mitochondrial DNA (mtDNA) damage further impairs mitochondrial function, compounding ROS production and leading to a vicious cycle of mitochondrial dysfunction and oxidative damage [19].

#### 2.1.3. Endoplasmic Reticulum Stress

The endoplasmic reticulum (ER) is a crucial organelle responsible for protein synthesis, folding, and modification within cells. When cells are subjected to various stressors, unfolded or misfolded proteins accumulate in the ER, leading to a state known as endoplasmic reticulum stress. This stress activates the unfolded protein response (UPR), a cellular mechanism aimed at restoring ER function by enhancing protein-folding capacity and degrading misfolded proteins. However, if the stress persists and the UPR fails to resolve the protein accumulation, ER dysfunction ensues, ultimately triggering cellular apoptosis [20]. During this process, the ER produces a large amount of ROS, exacerbating oxidative stress within the cell. Recent research suggests that ER stress plays a central role in triggering multiple molecular cascades linked to chronic diseases, including chronic pain, making it a potential therapeutic target [21].

### 2.2. Antioxidant Mechanism

**Figure 1:**
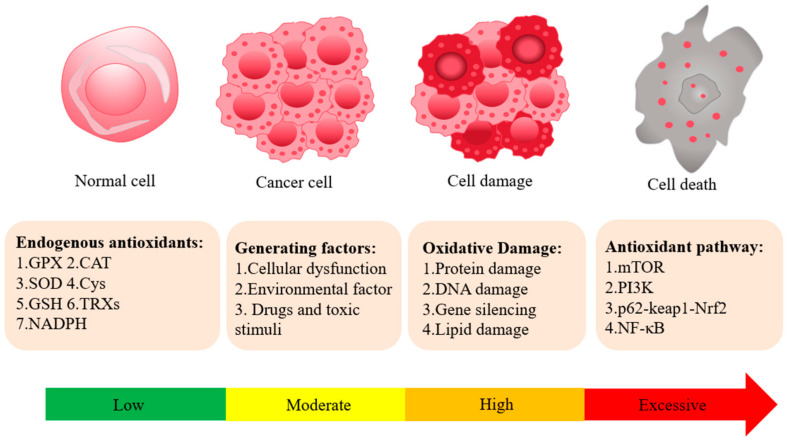
This figure depicts the mechanisms of cellular oxidative stress, highlighting the sources of reactive oxygen species (ROS), their damaging effects, and the antioxidant systems that neutralize them. It illustrates key enzymatic (e.g., superoxide dismutase [SOD], catalase [CAT], glutathione peroxidase [GPx], etc.) and non-enzymatic (e.g., glutathione [GSH], vitamins C and E, etc.) antioxidants that counteract ROS. The interplay between ROS production and antioxidant defenses is crucial for maintaining cellular redox homeostasis.

The human antioxidant system consists of both enzymatic and non-enzymatic components that work in tandem to protect cells from oxidative damage. When the body encounters external stimuli or internal metabolic dysregulation, this equilibrium is disrupted, resulting in the excessive production of reactive oxygen species (ROS) and free radicals, which cause cellular and tissue damage (Figure 1). The enzymatic antioxidant system includes key enzymes such as superoxide dismutase (SOD), catalase (CAT), and glutathione peroxidase (GPx), each of which plays a critical role in neutralizing reactive oxygen species (ROS) and maintaining cellular redox balance [22]. Among these, SOD has the highest antioxidant activity and is essential for converting superoxide radicals into less reactive species [23].

**Figure 2:**
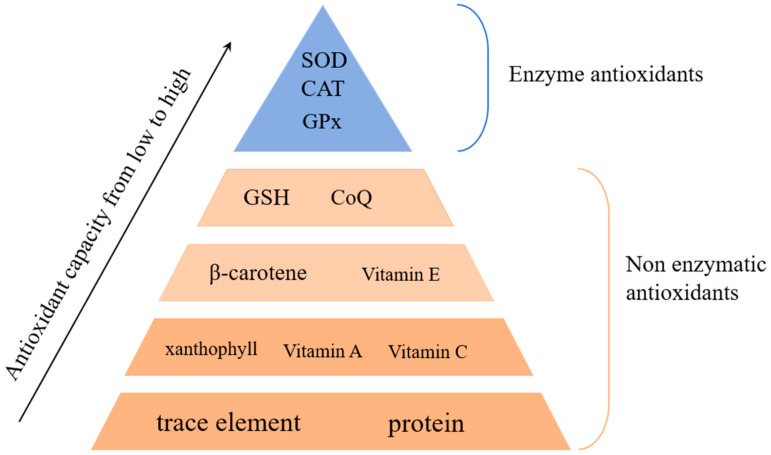
This figure compares the properties and roles of enzymatic and non-enzymatic antioxidant systems in cellular defense against oxidative stress. Enzymatic antioxidants, such as superoxide dismutase (SOD), catalase (CAT), and glutathione peroxidase (GPx), neutralize ROS in a stepwise manner. Non-enzymatic antioxidants, including glutathione (GSH), vitamins C and E, and plant-derived antioxidants, directly scavenge free radicals and provide additional protection.

The non-enzymatic antioxidant system includes molecules such as glutathione (GSH), vitamins C and E, melatonin, alpha-lipoic acid, and various plant-derived antioxidants like anthocyanins (Figure 2). These molecules scavenge free radicals, providing a secondary line of defense against oxidative stress [24]. For example, glutathione, a tripeptide widely found in cells, directly reacts with ROS to neutralize them. Vitamins C and E, which are well-known antioxidants, operate in aqueous and lipid environments, respectively, where they interact with and neutralize different types of ROS [25]. Together, these two systems create a comprehensive defense network, allowing cells to maintain homeostasis and prevent oxidative damage under stress conditions.

#### 2.2.1. Enzymatic Antioxidant System

Superoxide dismutase (SOD) is a crucial antioxidant enzyme that catalyzes the conversion of superoxide anions (O^2−^) into hydrogen peroxide (H_2_O_2_) and oxygen. As one of the more reactive oxygen species, O^2−^ has strong oxidative potential and can damage various biomolecules within cells. By converting O^2−^ into the relatively stable H_2_O_2_, SOD reduces cellular oxidative damage [26]. In humans, three primary SOD isoenzymes exist: copper–zinc superoxide dismutase (Cu/Zn SOD), located in the cytoplasm; manganese superoxide dismutase (Mn SOD), found in mitochondria; and extracellular superoxide dismutase (EC-SOD), which is present in extracellular fluids [27]. Each isoenzyme functions at distinct cellular sites, jointly providing a protective antioxidant shield against oxidative stress.

Catalase (CAT), primarily found in peroxisomes, decomposes H_2_O_2_ into water and oxygen. This rapid elimination of H_2_O_2_ prevents its accumulation, which otherwise could oxidize proteins, lipids, and DNA within cells [28]. The activity of CAT is regulated by various factors, including intracellular H_2_O_2_ concentration, iron ions, and hormone levels. Under oxidative stress, elevated H_2_O_2_ levels stimulate CAT activity, enhancing H_2_O_2_ clearance [29].

Glutathione peroxidase (GPx), a selenium-containing enzyme, is widely present in cell organelles like the cytoplasm, mitochondria, and nucleus. GPx uses reduced glutathione (GSH) to neutralize H_2_O_2_ and organic peroxides, converting them into water and non-toxic alcohols [30]. This process protects cells from oxidative damage. GPx activity is closely linked to cellular selenium levels, as selenium is an essential component of GPx; inadequate selenium reduces GPx activity, increasing cellular vulnerability to oxidative stress [31]. Recent studies have also identified GPx4 as a primary regulator of ferroptosis, highlighting its potential as a therapeutic target in diseases such as cancer and neurodegenerative disorders [32].

#### 2.2.2. Non-Enzymatic Antioxidant System

Glutathione (GSH) is a tripeptide (γ-glutamylcysteine glycine) widely present in cells with strong antioxidant capacity. GSH directly neutralizes ROS, converting them into harmless substances, such as water [33]. In addition, GSH serves as a substrate for glutathione peroxidase (GPx), which assists in clearing H_2_O_2_ and organic peroxides [34]. The synthesis of GSH requires several enzymes, with the most crucial enzymes being gamma-glutamylcysteine synthetase (γ-GCS) and glutathione synthase (GS) [35].

Vitamins C and E are well-studied antioxidants. Vitamin C, a water-soluble vitamin, has potent antioxidant properties, directly reacting with ROS to neutralize them. Studies have shown that vitamin C effectively manages type 2 diabetes and hypertension by enhancing the formation of anti-inflammatory and vasodilatory compounds, such as prostaglandin E1 (PGE1) and endothelial nitric oxide (eNO) [36]. Vitamin E, a fat-soluble vitamin mainly present in cell membranes, counters lipid peroxidation. By neutralizing free radicals in lipid membranes, vitamin E prevents oxidative damage, particularly to cell membranes [37]. Additionally, vitamin E’s antioxidant functions offer protection to nerve cells from oxidative damage, potentially delaying neurodegenerative processes like Alzheimer’s disease [38].

Carotenoids, natural pigments in plants, also possess strong antioxidant activity. Carotenoids can neutralize ROS and protect cells from oxidative damage. They also absorb UV radiation, shielding cells from UV-related harm. Common carotenoids include beta-carotene, lutein, and lycopene [39]. Beta-carotene, for example, converts into vitamin A, playing vital roles in vision, immunity, and cellular differentiation. Recent studies show beta-carotene can alleviate diabetic nephropathy via AMPK/SIRT1 autophagy pathways [40]. Lutein, primarily found in the retina, protects against retinal oxidative damage and has been linked to anti-cancer effects. Lycopene, another potent antioxidant, has demonstrated cardiovascular and cancer-protective effects [41].

### 2.3. The Role of AMPK-SIRT1-FOXO Pathway in Antioxidant Stress

The AMPK-SIRT1-FOXO pathway plays an important role in regulating cellular antioxidant defense and oxidative stress. The close interaction between the three molecules plays a crucial role in energy metabolism regulation and antioxidant defense. AMPK is an energy-sensing molecule that is activated when cells are in a state of energy depletion or metabolic stress, such as hunger or hypoxia [42]. Once activated, AMPK can promote SIRT1 activity. SIRT1 enhances the activity of FOXO family transcription factors (such as FOXO1 and FOXO3) by deacetylating them, thereby regulating the expression of various antioxidant-related genes [43]. FOXO is the main regulator of antioxidant genes, directly controlling the transcription of genes that upregulate various antioxidant enzymes (such as SOD, CAT, etc.) and proteins related to the oxidative stress response [44].

The AMPK-SIRT1-FOXO pathway affects enzymes responsible for ROS production through direct and indirect mechanisms. Among them, NADPH oxidase is one of the main sources of ROS production. The AMPK-SIRT1-FOXO pathway can reduce ROS generation by decreasing NOX expression or inhibiting its activity [45]. In addition, AMPK activates PGC-1α, which promotes mitochondrial biosynthesis and quality control, optimizes mitochondrial oxidative phosphorylation efficiency, and reduces ROS generation [46]. FOXO activates the BCL2L11 gene, thereby promoting mitochondrial autophagy, facilitating the clearance of damaged mitochondria, and reducing the sustained production of ROS [47].

The AMPK-SIRT1-FOXO pathway can also activate enzymatic and non-enzymatic antioxidant systems to clear ROS. FOXO3 upregulates the expression of SOD1 and SOD2, enhancing the decomposition of superoxide and reducing the accumulation of ROS [48]. FOXO1 and FOXO3 can induce CAT expression and promote the decomposition of H_2_O_2_ [49,50]. The AMPK-SIRT1-FOXO pathway enhances the clearance of lipid peroxides and H2O2 by regulating GPX expression and activity. FOXO activates key enzymes related to GSH synthesis, such as gamma-glutamylcysteine synthase, to increase intracellular GSH levels, while simultaneously enhancing the storage of non-enzyme antioxidants such as coenzyme Q and uric acid by regulating metabolic pathways [51].

## 3. AMPK

**Figure 3:**
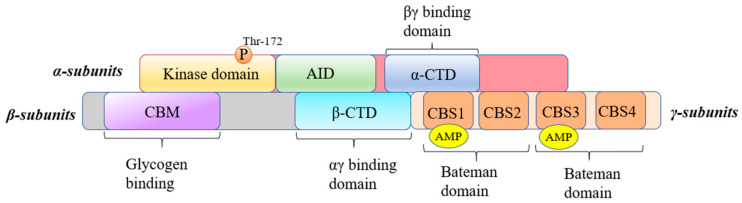
AMPK protein exists as a heterotrimeric complex, which is composed of an α-catalytic subunit, a β-regulatory subunit, and a γ-regulatory subunit. The N-terminus of the α-subunit contains a conserved kinase region that contains a conserved threonine (thr-172) site, and phosphorylation of this site is required for its kinase activity. It also includes an auto inhibitory domain (AID) and a region that binds to β-subunits and γ-subunits. The β-subunit contains two conserved regions—a middle glycogen-binding region (CHM) and a C-terminal-binding region with the other two subunits. The γ-subunit contains four tandem repeats of cystathionine-β-synthase (CBS) to form two Bateman domains, each of which can bind AMP or ATP.

Adenylate-activated protein kinase (AMPK) is a crucial molecule in regulating intracellular energy balance and homeostasis. It is expressed in various cell types, including hepatocytes, cardiomyocytes, adipocytes, and endometrial epithelial cells [52]. AMPK is a heterotrimeric complex that comprises a catalytic α subunit and two regulatory subunits, β and γ. The α subunit contains a kinase domain with a conserved phosphorylation site (Thr-172), which is essential for AMPK activation (Figure 3). AMPK monitors and balances nutrient availability against energy demands, thus coordinating anabolic and catabolic processes. The body maintains energy balance primarily in the form of adenosine triphosphate (ATP); when cellular ATP levels decrease, the AMPK pathway becomes activated to phosphorylate enzymes and proteins, resulting in ATP production and a reduction in ATP consumption [53].

AMPK was first described in a research report in 1973 [54], and its enzyme name was officially recognized by Munday et al. in 1988 [55], with further structural analysis provided by Bruce Kemp and colleagues in 1994 [56]. AMPK is a heterotrimeric complex that comprises a catalytic α subunit and two regulatory subunits, β and γ. To date, two types of α subunits, two types of β subunits, and three types of γ subunits have been identified [57]. The α subunit contains a kinase domain with a conserved phosphorylation site (Thr-172), which is essential for AMPK activation. Meanwhile, the β subunit provides a glycogen-binding site, allowing AMPK to bind to glycogen. The γ subunit is responsible for sensing changes in the AMP/ADP ratio, thereby regulating AMPK activation when cellular energy levels are low [58].

AMPK is vital in immune cell metabolism, particularly in macrophages, where it serves as a critical link between metabolism and inflammation and regulates the development of various diseases, including diabetes, obesity, and cancer [59]. For example, Xiao et al. demonstrated that 25-hydroxycholesterol (25HC) could reprogram immunosuppressive macrophages by altering AMPK activity and metabolism, identifying a novel metabolic target for tumor immunotherapy [60]. Additionally, Wang et al. found that the AMPK-TBC1D1 signaling pathway in macrophages regulates ROS synthesis, presenting a promising target for treating obesity and metabolic disorders [61].

AMPK also influences processes such as ferroptosis and pyroptosis, which are critical cell death pathways involved in various pathologies. For instance, Lu et al. discovered that empagliflozin could inhibit ferroptosis through the AMPK/Nrf2 pathway, providing a potential treatment for diabetic nephropathy [62]. Furthermore, Ai et al. found that mannose could inhibit GSDME-mediated pyroptosis by activating AMPK, revealing a mechanism that could be targeted in inflammatory diseases [63].

**Figure 4:**
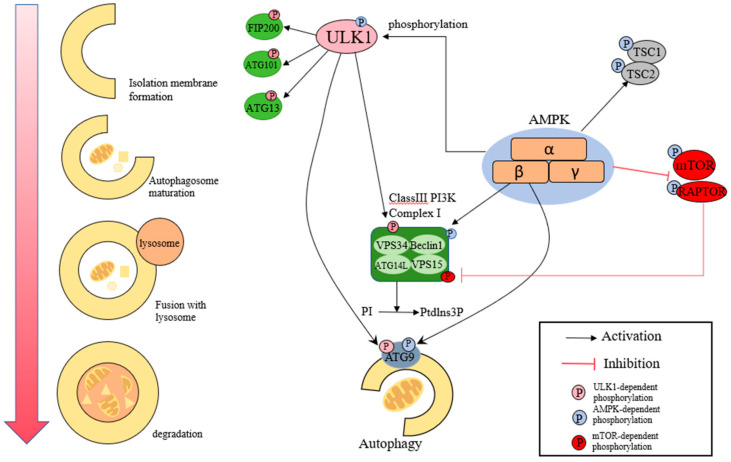
This figure illustrates the role of AMP-activated protein kinase (AMPK) in inducing autophagy. AMPK activation under conditions of energy depletion or oxidative stress inhibits the mTOR pathway, thereby initiating the autophagic process. Autophagy removes damaged organelles and proteins, maintaining cellular homeostasis and reducing oxidative stress. The figure highlights key components, including ULK1 activation and downstream lysosomal degradation. (AMPK: AMP-activated protein kinase. mTOR: mechanistic target of rapamycin. ULK1: Unc-51 like autophagy activating kinase 1. LC3: microtubule-associated protein 1 light chain 3.)

In the context of oxidative stress, AMPK activation can impact autophagy and apoptosis, which are essential for cellular survival under stress conditions (Figure 4). Studies have shown that AMPK activators modulate oxidative stress by balancing apoptosis and autophagy. For instance, metformin activates the AMPK/SIRT1/mTOR pathway, reduces oxidative stress, promotes autophagy, and inhibits apoptosis, thereby exhibiting therapeutic effects on non-alcoholic fatty liver disease [4]. Similarly, Hassan et al. reported that a combination of orlistat, metformin, and African mango seed extract could alleviate oxidative stress in mice fed a high-fat, high-carbohydrate diet by activating the AMPK/SIRT1/mTOR pathway [64]. Suski et al. found that AICAR, an AMPK activator, could inhibit inflammation and pro-apoptotic markers in the liver of apoE-knockout mice, reduce peroxisomal proteins, induce antioxidant autophagy, and alleviate atherosclerosis and non-alcoholic steatosis caused by oxidative stress in mice [65]. Fan et al. found that spermidine combined with exercise can induce autophagy, reduce apoptosis, and alleviate rat skeletal muscle atrophy caused by oxidative stress induced by galactose by activating the AMPK-FOXO3a signaling pathway [66].

**Figure 5:**
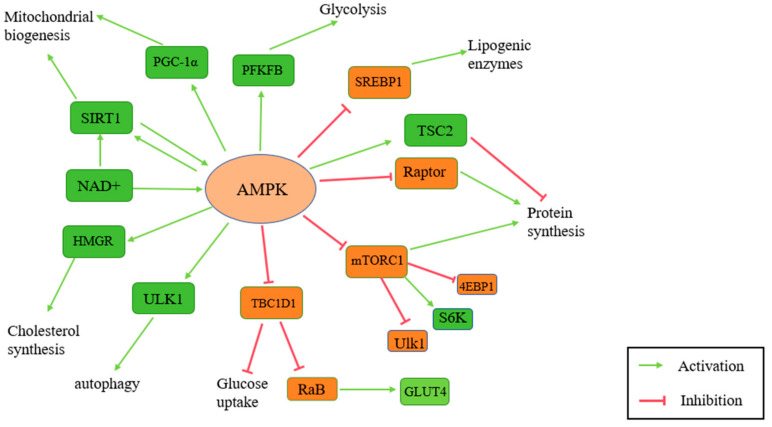
This figure illustrates the signaling networks regulated by AMP-activated protein kinase (AMPK). AMPK integrates energy-sensing and stress-response signals to modulate key processes, including lipid and glucose metabolism, oxidative stress reduction, autophagy activation, and mitochondrial function. Highlighted pathways include AMPK’s interactions with mTOR, PGC-1α, and FOXO, as well as its positive feedback loop with SIRT1 (AMPK: AMP-activated protein kinase. ACC: acetyl-CoA carboxylase. PGC-1α: peroxisome proliferator-activated receptor gamma coactivator 1-alpha. SIRT1: sirtuin 1. FOXO: Forkhead Box O. mTOR: mechanistic target of rapamycin. ULK1: Unc-51 like autophagy activating kinase 1).

AMPK is also localized in mitochondria and endoplasmic reticulum within endometrial epithelial cells, where it plays a role in maintaining endometrial receptivity. It is closely related to many signaling pathways (Figure 5). Inhibiting AMPK activity disrupts endometrial energy metabolism, potentially impairing embryo implantation. Ma et al. found that glucose utilization disorders in insulin-resistant mice led to endometrial glycogen imbalance, AMPK dysregulation, and eventually failed embryo implantation [67].

## 4. SIRT1

SIRT1, a member of the nicotinamide adenine dinucleotide (NAD+)-dependent class III histone deacetylase family, plays a pivotal role in regulating cellular physiology and energy homeostasis. It is widely expressed in mammalian cells across various organs—such as the liver, brain, kidneys, and skeletal muscle—and influences numerous critical processes, including inflammation, immunity, aging, apoptosis, pyroptosis, and cell proliferation, by targeting key proteins like AMPK, PGC-1α, CRTC2, and eNOS [68]. The core structure of SIRT1 comprises three domains—the catalytic domain, N-terminal, and C-terminal—that are essential for its lysine deacetylation function [69]. Depending on its localization, SIRT1 dynamically impacts cellular processes: in the cytoplasm, it modulates protein interactions, while in the nucleus, it regulates nuclear proteins, such as FOXO3a, and activates various antioxidant genes [45].

Resveratrol is a polyphenolic substance found in red wine. As an important SIRT1 activator, its mechanism of action involves two distinct pathways: SIRT1-dependent and SIRT1-independent, with multi-level regulation of AMPK activation. Firstly, resveratrol increases the bioavailability of NAD+, thereby activating SIRT1. Once activated, SIRT1 subsequently activates LKB1 (an AMPK upstream kinase) through deacetylation, which makes LKB1 more susceptible to phosphorylation and activation. The activated LKB1 then phosphorylates the catalytic subunit Thr172 of AMPK, directly promoting the activity of AMPK [70]. In addition to the SIRT1-dependent pathway, studies have shown that resveratrol can also directly or indirectly activate AMPK through an SIRT1-independent mechanism. Specifically, resveratrol directly acts on mitochondrial respiratory chain complex I, leading to an increase in the intracellular AMP/ATP ratio. This increase in AMP is a classic triggering mechanism for AMPK activation, which promotes AMPK activation by directly binding to the gamma subunit of AMPK [71]. This dual activation mode provides resveratrol with unique advantages in metabolic regulation and antioxidant stress.

Research on resveratrol has been ongoing for many years. As early as 2003, Howitz et al. found that resveratrol can reduce the Michaelis constant of SIRT1 for acetylated substrates and NAD(+), thereby improving cell survival by stimulating SIRT1-dependent deacetylation of p53 [72]. Later, Lagouge et al. found that, in yeast, resveratrol can simulate caloric restriction by stimulating SIRT2, which increases DNA stability and extends lifespan by 70% [73]. Kim et al. found that resveratrol can promote neuronal survival by activating SIRT1, reduce neurodegeneration in the hippocampus, and play an important role in preventing aging and neurodegenerative diseases [74]. Timmers et al. found that supplementing resveratrol activates AMPK, increases SIRT1 levels, and induces changes in calorie metabolism in obese individuals [75]. Price et al. found that SIRT1 mediates the process of resveratrol activating AMPK and improving mitochondrial function [76]. Park et al. found that resveratrol improves symptoms of age-related metabolic diseases by inhibiting cAMP phosphodiesterase and activating the CaMKKβ–AMPK–SIRT1 pathway [77]. In summary, resveratrol exhibits a unique mechanism of multi-level regulation of AMPK through both SIRT1-dependent and SIRT1-independent pathways. This mechanism demonstrates great potential in metabolic regulation, antioxidant stress, prevention of aging and neurodegenerative diseases, improvement in calorie metabolism in obese populations, and alleviation of symptoms of age-related metabolic diseases.

Caloric restriction (CR), recognized as one of the most effective strategies for prolonging lifespan and reducing age-related diseases, operates through SIRT1 activation. CR increases the NAD+/NADH ratio in cells, activating SIRT1 and promoting autophagy while regulating intracellular glucose homeostasis. For instance, Moreselli et al. found that CR and the SIRT1 activator, resveratrol, promote longevity by inducing SIRT1-mediated autophagy [78]. CR also activates AMPK by increasing the AMP/ATP ratio, which enhances NAD+ levels, indirectly activating SIRT1. In turn, SIRT1 activates AMPK by deacetylating its upstream kinase LKB1, forming a positive feedback loop crucial for energy homeostasis [79]. Moreover, Zhang et al. demonstrated that CR alleviates obesity in high-fat diet mice by activating the SIRT1/AMPK pathway [80].

CR also modulates other critical pathways, such as reducing metabolic rates, lowering insulin and IGF-1 levels [81], and downregulating the nutritional sensor mTOR. SIRT1 amplifies autophagic responses by deacetylating and activating TSC2, a major mTOR inhibitor, which is particularly significant under nutrient deprivation [82]. This coordinated regulation ensures robust autophagy during energy stress. For example, Liu et al. found that metformin alleviates excessive osteoclast differentiation and reduces mandibular osteoporosis in aging mice by correcting the dysregulated AMPK-mTOR-p53 pathway [83]. Similarly, Liao et al. discovered that aminophylline inhibits the progression of chronic renal failure by regulating SIRT1/AMPK/mTOR-mediated autophagy [84]. Ma et al. showed that bilobalide alleviates osteoarthritis in rats by inhibiting iNOS and COX-2 protein expression through the AMPK/SIRT1/mTOR pathway [85], while Shen et al. reported that paeonol improves hyperlipidemia and autophagy by targeting the Nrf2 and AMPK/mTOR pathways [86]. Furthermore, Yan et al. demonstrated that nicotinamide mononucleotide inhibits the proliferation of human liver cancer cells via the SIRT1/mTOR signaling pathway [87].

In addition to energy regulation, SIRT1 plays a key role in combating oxidative stress and inflammation. SIRT1 enhances cellular resilience to oxidative stress by regulating pathways involving AMPK, FOXO1, and FOXO3a, which mitigate ROS production and lipid peroxidation [88]. For example, Fan et al. observed that acupuncture-induced electrical stimulation reduces apoptosis and oxidative stress by activating the SIRT1/FOXO3a pathway in brain injury models [66]. Zhang et al. found that caffeine suppresses neuroinflammation and oxidative stress after spinal cord injury through the SIRT1/PGC1α/DRP1 pathway [89]. Additionally, Chen et al. demonstrated that Danggui Shaoyao Powder activates SIRT1 to exert anti-apoptotic, antioxidative stress, and ovary-protective effects in mice with premature ovarian failure [90]. Similarly, Zhao et al. found that ebony medicine extracts alleviate alcohol-induced oxidative stress and inflammation and regulate intestinal microbiota disorders through the SIRT1 pathway [91], while Zhu et al. showed that butanol reduces oxidative stress and apoptosis in treating brain injury caused by sepsis via SIRT1 activation [92].

**Figure 6:**
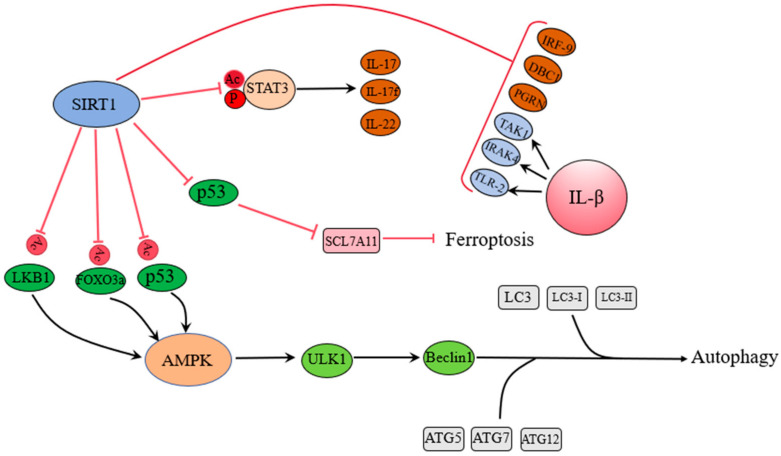
This figure illustrates the role of sirtuin 1 (SIRT1) in regulating autophagy, inflammation, and apoptosis. SIRT1 promotes autophagy by deacetylating key regulatory proteins and activating the AMPK-mTOR pathway. It suppresses inflammation by inhibiting NF-κB activity and enhances cellular survival by deacetylating p53 to reduce apoptosis. These mechanisms enable SIRT1 to protect cells against oxidative stress and maintain cellular homeostasis (SIRT1: sirtuin 1. NF-κB: nuclear factor kappa B. ULK1: Unc-51 like autophagy activating kinase 1. LC3: microtubule-associated protein 1 light chain 3. ROS: reactive oxygen species. AMPK: amp-activated protein kinase. mTOR: mechanistic target of rapamycin).

SIRT1′s interaction with p53 further exemplifies its role in cellular adaptation during stress (Figure 6). CR activates SIRT1, which deacetylates p53 to inhibit its pro-apoptotic activity, thereby promoting autophagy, enhancing stress resistance, and reducing oxidative damage [93]. For example, Wen et al. showed that galangin alleviates UV-induced cellular aging by enhancing SIRT1-mediated p53 deacetylation [94]. Similarly, Tang et al. demonstrated that iris suppresses ferroptosis in patients with type 1 diabetes via the SIRT1-p53 pathway, improving diabetes-induced cardiomyopathy [95]. Zeng et al. reported that dihydroquercetin alleviates acute liver failure by regulating the SIRT1/p53 axis to inhibit ferroptosis and mitochondrial-mediated apoptosis [96]. Furthermore, Zhou et al. observed that paeonol activates the SIRT1/p53/TRF2 pathway to inhibit vascular smooth muscle cell aging, potentially benefiting patients with atherosclerosis [97]. Interestingly, Metselaar et al. found that gemcitabine treatment degrades SIRT1, activating NF-κB and p53 to promote cell death, which has proven effective in treating atypical teratoid/rhabdoid tumors [98]. On the other hand, Jia et al. found that the overexpression of NOC4L binds to SIRT1, inhibits its expression, increases p53 acetylation, and promotes apoptosis, suppressing tumor growth [99]. Lo Cigno et al. demonstrated that EX527 inhibition of SIRT1 restores the transcriptional activity of K382-acetylated p53 in HPV+ cell lines, suppressing tumor development [100].

The mTOR pathway also interacts intricately with SIRT1 and p53 during energy stress. In the cytoplasm, p53 inhibits mTOR activity by interacting with TSC2, a key upstream regulator of mTOR [101]. This inhibition enhances autophagy, allowing cells to degrade and recycle damaged proteins and organelles for energy production. Such regulation is significant in disease treatment. For instance, Wang et al. found that huperzine enhances autophagy and alleviates LPS-induced acute lung injury via the p53/mTOR pathway [102]. Zhao et al. demonstrated that VD3 reduces high-fat diet-induced weight gain in mice by enhancing autophagy and regulating the PI3K/Akt/mTOR/p53 signaling pathway [103]. Additionally, Pi et al. reported that polysaccharides from Atractylodes macrocephala alleviate non-alcoholic fatty liver disease by inhibiting the p53/mTOR pathway [104], while Chen et al. found that miR-1972 promotes angiogenesis and alleviates rheumatoid arthritis through the same pathway [105].

**Figure 7:**
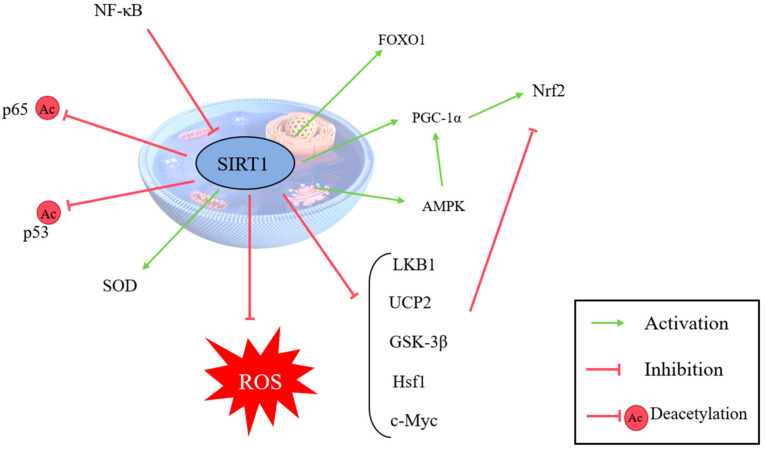
This figure illustrates the role of sirtuin 1 (SIRT1) in mitigating oxidative stress. SIRT1 activates antioxidant transcription factors (e.g., FOXO, Nrf2, etc.), promoting the expression of antioxidant enzymes such as superoxide dismutase (SOD) and catalase (CAT). It collaborates with AMPK to enhance mitochondrial biogenesis and function, reduces lipid peroxidation, and suppresses inflammation via NF-κB inhibition. Additionally, SIRT1 modulates p53 activity to limit apoptosis under stress conditions. (SIRT1: sirtuin 1. ROS: reactive oxygen species. SOD: superoxide dismutase. CAT: catalase. AMPK: AMP-activated protein kinase. NF-κB: nuclear factor kappa B.)

Finally, SIRT1′s role in endometrial diseases has also been extensively studied. Elevated SIRT1 expression in endometrial cancer has been linked to tumor growth, with Lin et al. and Asaka et al. showing that inhibiting SIRT1 suppresses tumor progression [106,107]. In endometriosis, SIRT1-mediated autophagy may provide protection against oxidative stress in endometrial cells, as Zhou et al. demonstrated, offering promising avenues for treatment [108]. In addition, SIRT1 is closely related to many signaling pathways (Figure 7).

## 5. FOXO

**Figure 8:**
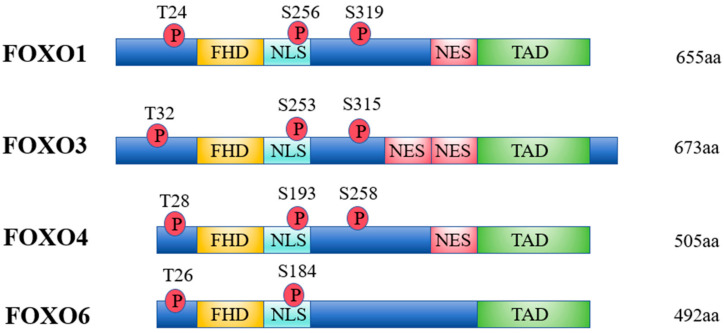
This figure illustrates the structural domains of Forkhead Box O (FOXO) transcription factors. Key domains include the DNA-binding domain (Forkhead Box), transactivation domain, and post-translational modification (PTM) sites for phosphorylation, acetylation, and ubiquitination. The nuclear localization signal (NLS) and nuclear export signal (NES) regulate FOXO’s cellular localization, enabling it to respond dynamically to oxidative stress and metabolic signals (FOXO: Forkhead Box O; PTM: post-translational modification; NLS: nuclear localization signal; NES: nuclear export signal).

Forkhead Box O (FOXO) proteins are a class of transcription factors characterized by a conserved Forkhead Box or winged-helix domain. The FOXO family in humans includes four main members, i.e., FOXO1, FOXO3, FOXO4, and FOXO6, each with distinct tissue distributions and functional roles (Figure 8). FOXO1 is primarily expressed in adipose tissue; FOXO3 in the heart, kidneys, brain, and ovaries; FOXO4 in skeletal muscles and the heart; and FOXO6 is uniquely located in the brain [109]. These transcription factors regulate critical cellular processes such as cell cycle control, apoptosis, energy metabolism, and oxidative stress responses. FOXO proteins maintain cellular homeostasis by modulating a range of mechanisms. In cell cycle regulation, they enhance the expression of cell cycle inhibitors like p27^Kip1^ and p21^Cip1^ while repressing CyclinD1 expression, thereby slowing cell proliferation [110]. They also regulate apoptosis by inducing the expression of genes such as BIM, FasL, and TRAIL, which promote programmed cell death under stress conditions [111]. In energy metabolism, FOXO factors modulate glucose and lipid metabolism genes, such as glucose-6-phosphatase (G6Pase) and pyruvate dehydrogenase kinase 4 (PDK4), which are essential for maintaining metabolic balance [112]. Under oxidative stress, FOXO proteins undergo post-translational modifications, such as phosphorylation, acetylation, and ubiquitination, which regulate their stability and activity. For instance, the ubiquitin–proteasome pathway degrades FOXO proteins, temporarily downregulating the antioxidant response [113]. However, FOXO transcription factors enhance the expression of antioxidant enzymes such as superoxide dismutase (SOD), catalase (CAT), and manganese superoxide dismutase (MnSOD), enabling cells to detoxify reactive oxygen species (ROS) [114]. The activity of FOXO proteins is intricately controlled by the PI3K-Akt signaling pathway and stress-responsive growth factors, allowing cells to balance survival and apoptosis under oxidative stress conditions [115].

FOXO1 plays a pivotal role in counteracting oxidative stress and regulating the aging process. It upregulates antioxidant enzymes such as SOD and glutathione peroxidase (GPx), helping eliminate free radicals and reduce oxidative damage [116]. For example, FOXO1 directly binds to the promoter region of the SOD gene, promoting its transcription and enabling the synthesis of SOD protein, which catalyzes the dismutation of superoxide anions (O_2_^−^·) into oxygen and hydrogen peroxide [117]. However, with age, FOXO1 expression significantly declines in key brain regions, such as the frontal, parietal, and occipital cortices and the hippocampus. This decline reduces its ability to activate antioxidant enzymes, including SOD, CAT, and GPx, leading to diminished ROS clearance [118]. As a result, the intracellular accumulation of ROS causes oxidative damage to cellular components such as membranes, proteins, and DNA. Superoxide anion radicals (O_2_^−^·) generate peroxynitrite anions (ONOO^−^) by reacting with nitric oxide (NO) and promote the formation of highly reactive hydroxyl radicals (·OH) through Fenton or Haber–Weiss reactions mediated by metal ions [119]. Moreover, a decrease in CAT and GPx activity hinders the removal of hydrogen peroxide (H_2_O_2_), leading to further ROS generation and oxidative damage. This cascade significantly contributes to aging and the onset of neurodegenerative diseases, such as Alzheimer’s disease (AD) and Parkinson’s disease (PD). For instance, in Alzheimer’s disease, decreased FOXO1 expression makes the brain more vulnerable to oxidative damage, exacerbating beta-amyloid accumulation and neuronal dysfunction [120]. In Parkinson’s disease, ROS-induced damage to dopaminergic neurons is intensified by FOXO1 deficiency, accelerating disease progression [121]. Additionally, FOXO1′s regulation of the Nrf2 signaling pathway further emphasizes its role in antioxidant defense. When FOXO1 levels drop, Nrf2 activity diminishes, impairing the upregulation of antioxidant enzymes and increasing oxidative stress [122].

FOXO3a is another essential transcription factor activated in cellular stress environments such as hypoxia and glucose deprivation. Under stress, FOXO3a translocates to the nucleus, where it regulates the transcription of genes involved in apoptosis, oxidative stress, and inflammation [123]. However, its activity is modulated by erythropoietin (EPO), a glycoprotein hormone that promotes red blood cell production and tissue protection. EPO prevents the nuclear translocation of FOXO3a by phosphorylating it via the PI3K/Akt signaling pathway. Phosphorylated FOXO3a is retained in the cytoplasm, losing its ability to activate stress-responsive genes, which reduces oxidative damage. EPO also mitigates ROS production by regulating FOXO3a-associated proteins, thereby protecting cells from oxidative stress-induced damage [124]. The protective effects of EPO are particularly relevant in brain endothelial cells, a critical component of the blood–brain barrier. Studies have shown that EPO helps maintain the integrity of the blood–brain barrier and reduces ischemic brain injury [125]. These findings highlight the potential of targeting FOXO3a in therapeutic strategies for conditions related to oxidative stress and inflammation.

FOXO proteins are integral to diseases caused by oxidative stress. In cancer, chronic oxidative stress activates signaling pathways that promote the survival and proliferation of cancer cells, contributing to tumor progression [126]. Decreased FOXO activity, especially of FOXO1 and FOXO3a, favors cell survival under oxidative stress. For instance, Engelman et al. demonstrated that PI3K/Akt pathway activation during tumorigenesis suppresses FOXO-mediated ROS detoxification, leading to elevated intracellular ROS levels and cancer development [127]. Similarly, Tothova et al. showed that hematopoietic stem cells from triple-knockout FOXO1, FOXO3, and FOXO4 mice exhibit increased ROS levels, linking reduced FOXO activity to oxidative stress and tumor progression [128].In non-cancerous conditions, FOXO proteins also play significant roles. Lu et al. demonstrated that chronic restraint-induced oxidative stress in sows leads to autophagy and apoptosis through FOXO1 activation. In pregnant mice under stress, FOXO1 activation in the β2-AR/FOXO1/NF-κB p65 pathway modulates inflammatory responses [115]. Furthermore, Chen et al. revealed that the long noncoding RNA LINC00963 inhibits renal fibrosis and oxidative stress in rats by activating the FOXO pathway, showcasing its therapeutic potential for kidney disease [129]. In Alzheimer’s models, Navarro et al. showed that a methanol extract from strawberries reduces beta-amyloid production and ROS in Caenorhabditis elegans through DAF-16/FOXO signaling, highlighting FOXO’s neuroprotective role [130]. Overall, FOXO proteins inhibit cancer progression by promoting apoptosis, DNA repair, cell cycle arrest, and antioxidant defenses. However, their age-related decline or suppression in diseases exacerbates oxidative stress, highlighting the need to target FOXO pathways for therapeutic interventions.

**Figure 9:**
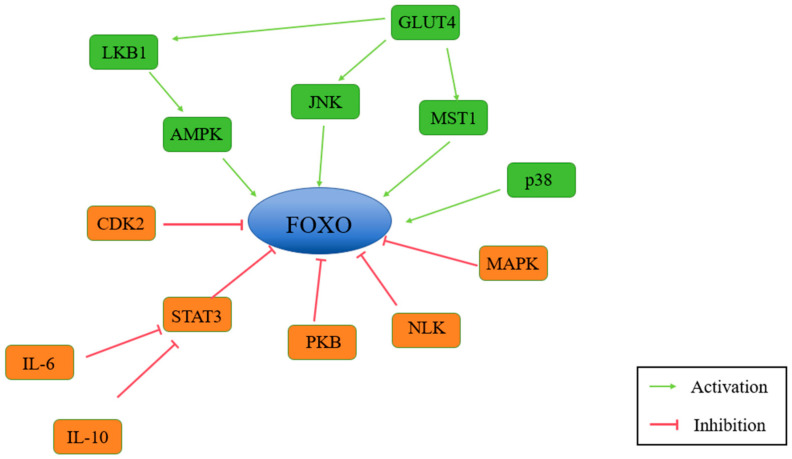
This figure illustrates the partial signaling pathways regulated by Forkhead Box O (FOXO) transcription factors. Upstream regulators include the PI3K/AKT pathway, which inhibits FOXO activity, and AMPK and SIRT1, which enhance FOXO activity through phosphorylation and deacetylation, respectively. FOXO mediates downstream effects such as antioxidant defense (e.g., SOD, CAT, etc.), autophagy activation, and apoptosis regulation. These pathways ensure cellular adaptation to oxidative and metabolic stress. (FOXO: Forkhead Box O; PI3K: phosphoinositide 3-kinase; AKT: protein kinase B; AMPK: AMP-activated protein kinase; SIRT1: sirtuin 1; SOD: superoxide dismutase; CAT: catalase; ROS: reactive oxygen species.)

In summary, FOXO proteins, particularly FOXO1 and FOXO3a, are essential transcription factors that regulate antioxidant defenses, stress responses, and apoptosis. In addition, FOXO is closely related to many signaling pathways (Figure 9). Their activity is intricately linked to oxidative stress and its pathological consequences, including cancer, neurodegenerative diseases, and other age-related conditions. A deeper understanding of FOXO regulation provides valuable insights into developing effective therapeutic strategies to combat oxidative stress and improve health outcomes.

## 6. AMPK-SIRT1-FOXO Pathway

The AMPK-SIRT1-FOXO pathway represents an intricate and highly coordinated cellular signaling network that is crucial for maintaining energy homeostasis and adapting to stress. This pathway establishes a direct link between cellular energy-sensing mechanisms and stress-responsive gene regulation, highlighting its multifaceted roles in cellular adaptation. It connects AMP-activated protein kinase (AMPK), a cellular energy sensor, with SIRT1, a NAD+-dependent deacetylase, and FOXO transcription factors, which mediate stress responses and gene expression changes. Together, these components work as a unified system to regulate key cellular processes, including energy metabolism, oxidative stress responses, mitochondrial quality control, and inflammation management.

At the core of the pathway is AMPK, which is activated in response to energy depletion. Cellular energy states are constantly monitored by AMPK, which becomes activated when the ATP/AMP/ADP ratio shifts due to stressors such as glucose deprivation, hypoxia, or physical exercise [131]. Once activated, AMPK initiates broad metabolic changes to conserve energy and restore balance. It promotes ATP-generating catabolic processes, such as glycolysis and fatty acid oxidation, while simultaneously suppressing ATP-consuming anabolic pathways, including protein, lipid, and glycogen synthesis. For example, in skeletal muscle cells, AMPK phosphorylates AS160, facilitating the translocation of glucose transporter GLUT4 to the cell membrane, enhancing glucose uptake under energy-deficient conditions [132]. Additionally, AMPK phosphorylates and inactivates acetyl-CoA carboxylase (ACC), a key enzyme in fatty acid synthesis, to promote mitochondrial beta-oxidation and reduce lipid accumulation [133].

SIRT1, on the other hand, links metabolic sensing to downstream regulatory pathways. As a deacetylase, it serves as a critical metabolic sensor, translating NAD+/NADH fluctuations into actionable cellular signals. This deacetylase responds to changes in the NAD+/NADH ratio, allowing it to function as a sensor of the cellular metabolic state [134]. Once activated, SIRT1 interacts closely with AMPK, forming a positive feedback loop. SIRT1 deacetylates and activates LKB1, a kinase responsible for AMPK activation, thereby amplifying energy-sensing mechanisms [70]. At the same time, AMPK influences SIRT1 activity by promoting NAD+ production, further enhancing SIRT1-dependent deacetylation processes [135]. Through this relationship, SIRT1 and AMPK jointly regulate downstream targets, such as PGC-1α, a key activator of mitochondrial biogenesis. This coordination not only improves mitochondrial efficiency but also reinforces metabolic flexibility in response to energy stress [68]. By phosphorylating and deacetylating PGC-1α, AMPK and SIRT1 work in tandem to stimulate mitochondrial function, improving cellular energy efficiency.

FOXO transcription factors, particularly FOXO1, FOXO3, and FOXO4, serve as critical downstream effectors of the AMPK-SIRT1 axis. These transcription factors integrate upstream signals and translate them into gene expression changes that promote cellular survival and stress adaptation. FOXO1, for instance, is activated by SIRT1-mediated deacetylation, enabling it to upregulate genes involved in gluconeogenesis, such as PEPCK and G6Pase, which are critical for maintaining glucose production during fasting or energy-deprived states [136]. Similarly, FOXO3 enhances the expression of genes encoding antioxidant enzymes, including catalase and superoxide dismutase (SOD), providing protection against oxidative damage caused by reactive oxygen species (ROS) [137]. These antioxidant effects highlight the FOXO factors’ essential role in managing oxidative challenges and preventing cellular dysfunction.

Another vital function of FOXO transcription factors is their regulation of autophagy, a cellular process essential for removing damaged organelles and maintaining mitochondrial quality. FOXO stimulates the expression of autophagy-related genes, enhancing lysosomal degradation pathways [138]. This action is pivotal for cellular quality control and complements AMPK and SIRT1, which act upstream to ensure energy homeostasis. Together, these processes ensure the removal of damaged mitochondria, reducing oxidative stress and preserving cellular function [139].

The AMPK-SIRT1-FOXO pathway is characterized by intricate feedback loops and cross-regulatory mechanisms. AMPK directly phosphorylates FOXO proteins, thus further enhancing their transcriptional activity, while SIRT1 fine-tunes FOXO activity through deacetylation. Such crosstalk ensures the pathway’s adaptability to diverse stressors and highlights its therapeutic potential. These interactions create a dynamic and adaptable regulatory system capable of responding to diverse cellular challenges, including energy depletion, oxidative stress, and inflammation.

### 6.1. Metabolic Diseases: Obesity and Diabetes

Metabolic diseases such as obesity and type 2 diabetes are characterized by chronic inflammation, insulin resistance, and impaired energy homeostasis. The AMPK-SIRT1-FOXO pathway mitigates these pathological processes by addressing dysregulated lipid and glucose metabolism, while simultaneously reducing oxidative stress and inflammation.

In obesity, excessive fat accumulation leads to chronic inflammation and metabolic dysfunction. AMPK activation plays a key role in counteracting these effects by regulating lipid metabolism. By phosphorylating and inactivating acetyl-CoA carboxylase (ACC), AMPK inhibits fatty acid synthesis and promotes mitochondrial beta-oxidation [140]. These mechanisms reduce lipid accumulation and enhance overall metabolic flexibility, preventing the further deterioration of metabolic health. SIRT1 complements these actions by suppressing the activity of PPARγ, a transcription factor that promotes adipocyte proliferation and differentiation. By inhibiting PPARγ, SIRT1 prevents excessive adipose tissue expansion and reduces inflammation associated with obesity [141].

In addition, SIRT1 regulates hypothalamic POMC neurons, which control appetite and satiety. By activating these neurons, SIRT1 reduces food intake and promotes energy balance [142]. FOXO transcription factors also contribute by regulating genes involved in fatty acid transport and lipid metabolism. For example, FOXO promotes the expression of fatty acid-binding proteins (FABPs), which facilitate fatty acid uptake and intracellular transport [143]. Additionally, FOXO factors help restore lipid homeostasis by reducing inflammation-associated metabolic stress, creating a synergistic effect with AMPK and SIRT1 [144].

In diabetes, the AMPK-SIRT1-FOXO pathway plays an essential role in maintaining glucose homeostasis. AMPK enhances glucose uptake in skeletal muscle by activating AS160 and promoting GLUT4 translocation to the plasma membrane [145]. Simultaneously, AMPK inhibits glycogen synthase and activates glycogen phosphorylase, ensuring a steady supply of glucose during energy stress [131]. These actions are particularly critical in mitigating hyperglycemia under insulin-resistant conditions. SIRT1 further enhances insulin sensitivity by deacetylating IRS proteins, reducing inhibitory serine phosphorylation, and promoting tyrosine phosphorylation [146]. This improves downstream insulin signaling, restoring glucose metabolism in insulin-resistant states. Meanwhile, FOXO1 regulates gluconeogenesis in the liver by activating genes such as PEPCK and G6Pase, ensuring glucose production during fasting or low-energy conditions [136]. Together, these mechanisms create a robust framework for re-establishing glucose and lipid balance in metabolic diseases.

### 6.2. Cardiovascular Diseases: Atherosclerosis and Ischemia–Reperfusion Injury

Cardiovascular diseases, including atherosclerosis and ischemia–reperfusion injury, are driven by oxidative stress and chronic inflammation. The AMPK-SIRT1-FOXO pathway offers significant protective effects in these conditions by regulating lipid metabolism, reducing inflammation, and preserving vascular integrity.

In atherosclerosis, oxidative stress induces the oxidation of low-density lipoprotein (LDL) into oxidized LDL (ox-LDL), which accumulates in the vascular wall. This triggers inflammatory responses and promotes the formation of foam cells, which contribute to plaque development [147]. AMPK counters these effects by inhibiting the NF-κB pathway, a key driver of vascular inflammation. AMPK phosphorylates and inhibits IKK, preventing NF-κB activation and reducing the production of pro-inflammatory cytokines such as TNF-α, IL-6, and MCP-1 [148]. This anti-inflammatory action also helps limit foam cell formation and vascular dysfunction. Additionally, AMPK enhances cholesterol efflux from vascular cells by activating ABCA1, a transporter that facilitates the removal of cholesterol to high-density lipoprotein (HDL) [149]. This reduces lipid accumulation in vascular walls and delays plaque formation.

SIRT1 complements these actions by deacetylating NF-κB, further suppressing its transcriptional activity and limiting endothelial inflammation. Furthermore, SIRT1 activates liver X receptor (LXR), promoting reverse cholesterol transport and decreasing lipid deposition in vascular tissues [150]. FOXO transcription factors contribute by enhancing endothelial nitric oxide synthase (eNOS) activity, thereby increasing nitric oxide (NO) production [151]. This NO-mediated vasodilation improves blood flow and mitigates oxidative damage in vascular endothelial cells, enhancing the overall cardiovascular health.

In ischemia–reperfusion injury, the sudden restoration of blood flow following ischemia generates excessive ROS, leading to mitochondrial damage and cell death. AMPK mitigates this damage by inhibiting mTOR signaling, preventing apoptosis, and promoting autophagy to clear damaged organelles [86]. SIRT1 deacetylates p53, suppressing its pro-apoptotic activity, and enhances mitochondrial biogenesis via PGC-1α activation [72]. Activation of FOXO can upregulate Bax and CCND1 and lyse caspase 8, downregulate apoptosis-related proteins, and alleviate myocardial ischemia–reperfusion injury [152]. Together, these interactions form a coordinated response to ischemic damage, reducing oxidative injury and promoting tissue regeneration.

### 6.3. Neurodegenerative Diseases: Alzheimer’s Disease and Parkinson’s Disease

Neurodegenerative diseases, including Alzheimer’s disease (AD) and Parkinson’s disease (PD), are characterized by oxidative stress, protein aggregation, and inflammation. The AMPK-SIRT1-FOXO pathway protects against these conditions by enhancing protein clearance, reducing ROS, and mitigating inflammation.

In AD, ROS oxidize amyloid-beta (Aβ) protein, increasing its neurotoxicity and promoting its aggregation into oligomers and plaques. These aggregates activate microglia and astrocytes, releasing inflammatory mediators that exacerbate neuronal damage [153]. AMPK reduces Aβ production by promoting the non-amyloidogenic cleavage of amyloid precursor protein (APP) [154]. SIRT1 activates autophagy through ULK1, enabling the clearance of Aβ aggregates. This process not only reduces plaque burden but also enhances neuronal survival and synaptic function [155]. FOXO transcription factors enhance lysosomal degradation pathways, further reducing Aβ accumulation and preserving neuronal function [156].

In PD, alpha-synuclein aggregation disrupts dopaminergic neurons, impairing motor function. AMPK reduces alpha-synuclein aggregation by phosphorylating the protein, altering its conformation, and reducing its toxicity [157]. These phosphorylation events also facilitate alpha-synuclein clearance via autophagic pathways. SIRT1 and FOXO enhance autophagy and lysosomal pathways to clear alpha-synuclein aggregates, protecting neurons from oxidative damage [158]. Such synergistic actions help preserve dopaminergic neuronal health and slow the progression of PD.

### 6.4. Cancer

The AMPK-SIRT1-FOXO pathway exhibits dual roles in cancer progression, acting as both a tumor suppressor and a promoter of cancer cell survival. On the one hand, the pathway restricts tumor growth by depriving cancer cells of energy and inducing apoptosis. AMPK inhibits glycolysis and fatty acid synthesis, curbing the metabolic flexibility of cancer cells, which is essential for their rapid proliferation. Additionally, AMPK promotes apoptosis by regulating Bcl-2 family proteins, reducing anti-apoptotic Bcl-2 activity, and enhancing pro-apoptotic Bax activation [159].

Conversely, the pathway supports cancer cell survival under stress by promoting DNA repair and metabolic flexibility. AMPK activates ataxia telangiectasia mutated (ATM) protein, initiating homologous recombination repair and non-homologous end joining [160]. SIRT1 deacetylates p53, suppressing its pro-apoptotic activity, while FOXO upregulates DNA repair proteins such as BRCA1, maintaining genomic stability in cancer cells [93,161]. In hypoxic tumor microenvironments, AMPK and SIRT1 enhance mitochondrial function and metabolic adaptation, allowing cancer cells to survive energy stress [162]. These dual roles complicate efforts to target the AMPK-SIRT1-FOXO pathway in cancer therapy. For instance, while inhibiting this pathway could suppress tumor growth, it may also reduce the cancer cell’s ability to respond to chemotherapeutic stress, necessitating context-dependent therapeutic strategies.

### 6.5. Aging

Aging is driven by cumulative oxidative damage, mitochondrial dysfunction, and chronic inflammation induced by the senescence-associated secretory phenotype (SASP). SASP involves the secretion of inflammatory cytokines such as IL-6, IL-1β, and TNF-α, which accelerate tissue degradation [163]. The AMPK-SIRT1-FOXO pathway delays aging by mitigating oxidative stress, stabilizing energy metabolism, and reducing inflammation.

AMPK inhibits mTOR, reducing cellular growth and preserving metabolic stability. This inhibition also activates autophagy, which plays a crucial role in clearing damaged organelles and maintaining cellular quality over time [164]. SIRT1 deacetylates NF-κB, suppressing SASP-related cytokine production, and enhances mitochondrial function through PGC-1α. By maintaining mitochondrial quality, SIRT1 reduces the energy burden associated with aging cells, slowing their functional decline [165]. FOXO transcription factors regulate SASP genes, preserving tissue structure and slowing aging. FOXO also upregulates antioxidant enzymes, such as superoxide dismutase (SOD), protecting cells from cumulative oxidative damage, which is a hallmark of aging [166].

The dynamic interplay between AMPK, SIRT1, and FOXO highlights their pivotal role in regulating longevity. Therapeutic interventions aimed at this pathway hold the potential to not only delay aging but also ameliorate age-associated diseases, such as neurodegenerative disorders, cardiovascular diseases, and metabolic dysfunctions.

The AMPK-SIRT1-FOXO pathway serves as a central regulator of cellular homeostasis, orchestrating an intricate network of metabolic, oxidative, and inflammatory processes to protect against metabolic disorders, cardiovascular diseases, neurodegeneration, cancer, and aging. Its ability to dynamically adapt to a wide range of cellular stressors underscores its critical role in maintaining physiological balance and resilience. This makes it a promising target for therapeutic intervention, not only to mitigate disease progression but also to promote healthspan and longevity through enhanced cellular efficiency and stress resistance.

## 7. Conclusions

The AMPK-SIRT1-FOXO pathway plays a pivotal role in regulating oxidative stress and maintaining cellular energy balance. Through its coordinated activation, the pathway not only promotes mitochondrial quality control, antioxidant defense, and metabolic flexibility but also supports cellular adaptation to diverse stress conditions, which are essential for protecting cells against oxidative damage. The pathway’s central role in diseases such as cardiovascular disorders, diabetes, neurodegenerative conditions, and cancer underscores its importance as a therapeutic target.

This review highlights how AMPK activation, through energy sensing, stimulates SIRT1 via increased NAD+ levels [70], forming a positive feedback loop that enhances FOXO-mediated antioxidant gene expression [43]. Key experimental evidence has demonstrated the effectiveness of natural compounds, such as resveratrol, and lifestyle interventions, including caloric restriction, in activating this pathway [71,78]. These interventions not only improve cellular resistance to oxidative damage but also show significant potential in mitigating disease progression. Furthermore, studies show that therapeutic interventions targeting this pathway can improve mitochondrial function, reduce inflammation, and regulate autophagy, collectively offering novel approaches for treating oxidative stress-related diseases.

Future research should focus on clarifying the tissue-specific mechanisms of the AMPK-SIRT1-FOXO pathway and exploring its crosstalk with other cellular signaling networks. In particular, unraveling how this pathway integrates with broader cellular processes will provide insights into its therapeutic potential. Developing selective modulators that target individual components of this pathway with minimal side effects could advance therapeutic applications. Additionally, combining pathway modulation with existing therapies, such as anti-inflammatory agents and immunotherapy, may enable the development of synergistic and integrative treatment strategies.

In conclusion, the AMPK-SIRT1-FOXO pathway offers a comprehensive framework for addressing oxidative stress and its associated diseases. By leveraging insights into its regulatory mechanisms, researchers can develop targeted interventions to enhance antioxidant defenses, improve metabolic health, and extend healthspan. These efforts hold the potential to translate foundational research into clinically meaningful outcomes.
